# Growing relevance of reports of adolescent cyberbullying victimization among adult outpatients

**DOI:** 10.1186/s12889-023-16342-y

**Published:** 2023-08-08

**Authors:** Benjamin Iffland, Lena M. Bartsch, Hanna Kley, Frank Neuner

**Affiliations:** Department of Psychology, Bielefeld University, Postbox 100131, 33501 Bielefeld, Germany

**Keywords:** Cyberbullying victimization, Prevalence rates, Clinical population, Outpatient clinic

## Abstract

In the general population, prevalence rates of cyberbullying victimization have continuously increased over the past decades. However, the extent to which these increasing numbers affect clinical populations seeking treatment in outpatient services remains an open question. The present study sought to examine whether the increase of cyberbullying victimization is also reflected by increased reports of cyberbullying victimization in a clinical outpatient population. In addition, we assessed the incremental contribution of experiences of cyberbullying in the prediction of psychological symptoms when controlling for histories of childhood maltreatment and offline peer victimization. For this purpose, we analyzed routine data from *N* = 827 outpatients who had sought treatment at a University outpatient clinic for psychotherapy between 2012 and 2021 in a cross-sectional study design. Analyses showed that 8.3% of the patients born in the years 1980 to 2002 indicated the experience of cyberbullying victimization in their adolescence. The rate of reported cyberbullying victimization increased from 1 to 3% in patients born in the years 1980 to 1987 to 24% in patients born in the year 2000. A logistic regression revealed that patients born in the years 1995–2002 were up to nineteen times as likely to report cyberbullying victimization as patients born in the years 1980–1982. In addition, hierarchical multiple regression analyses indicated that cyberbullying victimization significantly accounted for an incremental proportion of variance (1%) in the prediction of psychological symptom distress after controlling for child maltreatment and offline peer victimization. In conclusion, this retrospective survey indicates an increase of the clinical relevance of cyberbullying victimization both in frequency of and potential contribution to etiology. Raising attention to cyberbullying in clinical care and research seems to be justified and warranted.

## Introduction

The availability and use of online applications by adolescents have increased significantly in recent years [1]. In terms of content, adolescents predominantly use the internet for the purpose of communication [1]. Hence, social media sites, with all their benefits and risks, have turned into primary locations of social life [2]. Adolescents use social media as a playground to create and form their identity. That said, such spaces are not fully benign. Self-disclosure via communication technologies (e.g., posting pictures) can have long-term detrimental effects due to their digital footprint [3]. Particularly, social media use elevates young people’s risk for involvement in cyberbullying victimization [4]. Cyberbullying victimization is characterized by experiencing behaviors that repeatedly communicate hostile or aggressive messages intended to inflict harm or discomfort on others which are performed through electronic or digital media by individuals or groups [5].

The phenomenon of cyberbullying is conceptually related to offline bullying, in particular relational bullying [6], since it involves damaging the reputation of others by spreading rumors, sending threatening or hurtful messages, or distributing inappropriate photos or videos. However, there are some specific characteristics of cyberbullying that may justify considering it as distinct phenomenon. Bullying typically involves intentionality, repetition, and power imbalance. These aspects have different implications in cyberspace [7]. A power imbalance can be produced by media skills (e.g., creating a fake profile) or the ability to remain anonymous. Repetition can either refer to new actions or to repeated confrontations via the number of views by the victim or others [8]. In addition, cyberbullying allows anonymity of the perpetrators who do not need to fear social or legal accountability, the increased breadth of the potential audience in social media, and the difficulty of escaping cyberbullying that typically extends beyond the school context [7]. Furthermore, the victim’s reaction is not directly visible in the short term, which can lead to disinhibition effects that may increase the tendency to behave more hurtfully online than in reality [9].

In recent years, the rising availability of online applications has been accompanied by their growing misuse [4]. Jones et al. [10] described a significant increase in online harassment victimization among 10- to 17-year-olds by 5% points between the years 2000 and 2010, leading to a one-year prevalence of 11% in 2010 in the United States. Similarly, Kessel Schneider et al. [11], reported an increase by 6% points from 2006 (15%) to 2012 (21%) using the MetroWest Adolescent Health Survey. Recently, Kliem et al. [12] compared 6-month prevalence rates of cyberbullying victimization of ninth graders in the years 2013, 2015, and 2017. The authors reported significantly increasing values of 3.8% in 2013, 4.7% in 2015, and 6.1% in 2017. Thus, reported rates of cyberbullying victimization have been increasing [10–12] or at least remaining consistent [13] over the past decade.

Recently, researchers have argued that cyberbullying victimization causes negative effects similar to offline victimization [14]. Cyberbullying victimization has been shown to be particularly associated with internalizing problems such as depression [15, 16], low self-esteem and loneliness [17], anxiety and social anxiety [18, 19], and somatization [20]. Additionally, suicidality has emerged as the most concerning consequence of cyberbullying victimization [21, 22]. Further, a link between cyberbullying victimization and externalizing behaviors, such as increased alcohol and drug use or delinquent behavior, has also been reported [23, 24]. Although most of the presented research is limited to investigations in student populations (e.g., [25, 26]), a recent retrospective study indicated that negative consequences of cyberbullying victimization persist into adulthood [27].

Despite findings on increasing prevalence rates and a broad range of negative short-term and long-term consequences in student populations, there remains a limited body of knowledge regarding the prevalence rates and psychopathological outcomes of cyberbullying victimization in clinical populations [28]. Kranhold et al. [29] presented prevalence rates of different types of bullying using an outpatient sample of children and adolescents (average age 12 years old). While 24.5% of the outpatients reported bullying experiences in the past six months, 3.4% experienced cyberbullying, and only a small number of adolescents reported cyberbullying experiences only without experiences of offline bullying. However, it remains unclear to what extent older, e.g., adult, patient populations were affected by cyberbullying in their adolescence and what consequences these experiences may have had on their current symptomatology.

The high overlap of online and offline bullying in this study indicates that, despite some conceptual differences, cyberbullying is not a qualitatively distinct phenomenon but an additional method of relational bullying [30]. Several authors have argued that the clinical relevance of cyberbullying is overrated [31, 32] and does not provide additional explanatory value for the prediction of negative mental health outcomes [33]. However, some studies found that cyberbullying contributes to psychological symptoms in community populations over and above the influence of offline victimization (e.g., [19, 34, 35]). Van Geel et al. [36] observed that cyberbullying is more strongly associated with suicidal ideation than offline bullying. However, it remains unclear to what extent these findings can be translated to clinical populations to document their relevance for psychological disorders.

The aim of the present study was two-fold. First, we aimed to investigate whether the documented increase of cyberbullying victimization present in student populations [10–12] is reflected by increased prevalence rates of cyberbullying victimization in a clinical outpatient population over a period of 20 years with adult patients born in the years 1980–2002. We assumed that the likelihood of reporting cyberbullying victimization increased over time such that younger patients are more likely to report cyberbullying victimization. Second, we aimed to examine differential unique contributions of various forms of child maltreatment and relational peer victimization to psychopathology in a clinical outpatient population. Given the fact that bullying is associated with experiences of child maltreatment by caretakers (e.g., [37–40]) we assessed the incremental contribution of experiences of cyberbullying victimization in the prediction of psychological symptoms when controlling for histories of childhood maltreatment as well as offline peer victimization. For exploratory purposes, we also compared psychopathological profiles of those who experienced cyberbullying victimization in adolescence and those who did not when controlling for experiences of child maltreatment and traditional relational peer victimization. In particular, we expected that victims of cyberbullying would exhibit higher levels of psychopathology than individuals who did not experience cyberbullying.

## Methods

### Study setting

The present study utilized data from the outpatient clinic for psychotherapy at Bielefeld University. In the outpatient clinic, patient data were routinely collected over the course of treatment to learn more about causal or maintaining factors of mental illnesses. The dataset contains data from 2012 to the beginning of the year 2021.

### Participants

Data for this cross-sectional study were obtained from 1,325 treatment-seeking outpatients who completed various self-report questionnaires for clinical and scientific purposes before beginning psychotherapeutic treatment. As not all patients in the age range studied were at risk of experiencing cyberbullying in their youth, a sample of *N* = 832 patients was extracted, including only those born 1980 and later. This decision was based on theoretical and practical considerations. An increase in the availability and use of communication technologies is assumed to have occurred around the year 2000 at the earliest [1]. Cyberbullying victimization during the ages of 13 to 18 years was retrospectively reported. Therefore, patients who were at least 13 years old in 1993 (18 years old in 1998 respectively) were included in the analyses as the upper age limit of the inclusion range. Additionally, five participants were excluded from analyses because data on cyberbullying victimization was missing, resulting in a final sample size of *N* = 827. All patients provided their written consent for using the anonymized data for scientific purposes. The study procedure was approved by the Ethics Committee of Bielefeld University (protocol number: EUB-2022-274). Demographic characteristics and means from the assessments are presented in Table 1.

**Table 1:** Subject characteristics and mean values on assessments (N = 827)

	*Total sample* *(N = 827)*	*Patients reporting cyberbullying* *(n = 69)*	*Patients not reporting cyberbullying* *(n = 758)*	*p*
Age at time of assessment, *M* (*SD, range)*	25.72 (4.57, 18–39)	22.90 (3.47, 18–32)	25.98 (4.58)	< 0.001
Gender, % female (*n*)	60.3 (499)	71.0 (49)	59.4 (450)	
Traditional relational peer victimization^1^, *M* (*SD*)	11.58 (7.91)	18.08 (10.38)	10.99 (7.37)	< 0.001
Cyberbullying victimization², % experienced (*n*)	8.3 (69)			
Child maltreatment³, *M* (*SD*)	41.71 (15.28)	47.15 (17.32)	41.22 (15.00)	0.002
Emotional abuse, *M* (*SD*)	10.13 (5.11)	11.65 (5.51)	9.99 (5.05)	0.010
Emotional neglect, *M* (*SD*)	11.66 (5.04)	12.46 (5.40)	11.59 (5.00)	0.176
Physical abuse, *M* (*SD*)	6.54 (3.24)	7.25 (3.92)	6.48 (3.17)	0.118
Physical neglect, *M* (*SD*)	7.31 (3.00)	8.22 (3.43)	7.23 (2.95)	0.009
Sexual abuse, *M* (*SD*)	6.07 (3.33)	7.45 (5.39)	5.95 (3.05)	0.025
Brief Symptom Inventory				
Somatization, *M* (*SD*)	5.84 (5.09)	8.85 (5.72)	5.57 (4.95)	< 0.001
Obsessive-compulsivity, *M* (*SD*)	10.11 (5.11)	12.51 (4.27)	9.90 (5.13)	< 0.001
Interpersonal sensitivity, *M* (*SD*)	7.06 (4.11)	9.32 (3.76)	6.86 (4.08)	< 0.001
Depression, *M* (*SD*)	10.27 (5.74)	12.12 (4.82)	10.10 (5.79)	0.002
Anxiety, *M* (*SD*)	7.91 (5.02)	10.71 (5.15)	7.66 (4.93)	< 0.001
Hostility, *M* (*SD*)	6.07 (3.98)	8.18 (3.92)	5.88 (3.93)	< 0.001
Phobic anxiety, *M* (*SD*)	4.71 (4.46)	7.04 (5.33)	4.50 (4.31)	< 0.001
Paranoid ideation, *M* (*SD*)	5.95 (4.49)	8.74 (4.54)	5.70 (4.41)	< 0.001
Psychoticism, *M* (*SD*)	5.75 (4.05)	7.44 (4.22)	5.60 (4.00)	< 0.001
General Severity Index, *M* (*SD*)	1.31 (0.63)	1.74 (0.54)	1.27 (0.63)	< 0.001

Note: ^1^Assessed by the Fragebogen zu belastenden Sozialerfahrungen [Adverse Social Experiences Questionnaire] (FBS; Sansen et al., 2013), the sum score has been calculated under exclusion of two items not measuring relational victimization and items measuring cyberbullying victimization; ²Refers to one item of the FBS (adolescence subscale); ³Assessed by the Childhood Trauma Questionnaire (CTQ; Wingenfeld et al., 2010). Significance levels are not corrected for multiple testing.

### Instruments

Experiences of peer victimization were assessed using the *Fragebogen zu belastenden Sozialerfahrungen* [Adverse Social Experiences Questionnaire] (FBS; [41]). This self-report questionnaire consists of 22 items describing aversive social situations like rejection, exclusion, being laughed at, insulted, and teased by peers (e.g., “I was excluded from games or activities by other children or adolescents,” “I have been laughed at in the presence of other children”). For each situation, respondents were asked whether or not they experienced this situation during childhood (age 6–12) or adolescence (age 13–18). Sum scores of “Yes” responses across both age periods are calculated and range from 0 to 22 for a Childhood scale and an Adolescence scale and from 0 to 44 for a total score. The total score of the FBS presented with stability over a 20-month period (*r* = .89) [41]. Construct validity was confirmed through correlations between the FBS and psychological symptom distress as well as social anxiety. For the purposes of the present study, one item assessing physical peer victimization (“Other children or adolescents hit me or attacked me.”) was excluded from analyses in order to examine relational peer victimization exclusively. Cyberbullying victimization was assessed using the item of the FBS “Other children or adolescents have spread lies or rumours about me on the internet [e.g., in chat programmes] or published embarrassing videos of me”) from the adolescence scale, which refers to the concept of denigration [42]. Therefore, for the assessment of traditional forms of peer victimization, a sum score of the childhood and adolescence scales of the FBS without using the items assessing physical and cyberbullying victimization was used in the following analyses, with possible scores ranging from 0 to 40. In the present sample, the internal consistencies of the original and the modified FBS were high with Cronbach’s α = 0.91 (sum score), α = 0.89 (childhood scale), and α = 0.87 (adolescence scale).

Child maltreatment was assessed using the German Version of the Childhood Trauma Questionnaire (CTQ; [43]). The self-report questionnaire consists of a total of 28 items assessing all common types of childhood maltreatment (emotional abuse, emotional neglect, physical abuse, physical neglect, and sexual abuse) that have occurred before the age of 18. The items are rated from 1 (never true) to 5 (very often true) with a possible range of subscale scores from 5 to 25. In the present study, dimensional sum scores for each CTQ subscale were used in the statistical analyses. The psychometric properties of the German version are similar to the original version, and it has been shown to be a reliable and valid screen for childhood maltreatment. In the current sample, internal consistency was excellent for all items (Cronbach’s α = 0.93). Similarly, internal consistency of the sexual abuse, emotional neglect, emotional abuse, and physical abuse subscales was good to excellent (all Cronbach’s α’s > 0.84). The physical neglect subscale, however, showed only a questionable internal consistency (Cronbach’s α = 0.65). Accordingly, the subscale presented with a weak internal consistency in comparison to the other subscales and was highly correlated with the other CTQ subscales in a prior validation study [44]. As a consequence, the CTQ physical neglect subscale was not included in the following statistical analyses.

Psychopathology and psychological symptom and distress levels in general were measured using the German version of the Brief Symptom Inventory (BSI; [45]). The self-report questionnaire consists of 53 items, producing nine primary symptom dimensions (somatization, obsessive-compulsive symptoms, interpersonal sensitivity, depression, anxiety, hostility, phobic anxiety, paranoid ideation, and psychoticism). Additionally, three global indices measure general psychological symptom distress: the Global Severity Index (GSI); the Positive Symptom Total (PST); and the Positive Symptom Distress Index (PSDI). The items are rated on a 5-point Likert scale, ranging from 0 (not at all) to 4 (extremely), and refer to the past 7 days. In the present study, the GSI and the nine symptom dimensions were used to indicate participants’ psychological symptom distress. The GSI showed high internal consistency in the current sample (Cronbach’s α = 0.95). Internal consistency for the nine symptom dimensions ranged between Cronbach’s α = 0.67 (psychoticism) and Cronbach’s α = 0.84 (depression) in the present sample.

### Statistical analyses

All statistical analyses were performed using the Statistical Package for the Social Science (SPSS) 28. Due to sample size as well as less than 1% missing values per variable, listwise deletion was used for missing data. Preliminary analyses included Pearson correlations to examine associations between all child maltreatment subtypes, traditional relational peer victimization, cyberbullying victimization, and the different symptom dimensions measured by the BSI.

Since the prevalence rates of cyberbullying victimization varied greatly depending on the number of cases per year of birth, moving averages taking the averages of three consecutive years of birth were calculated for a descriptive comparison of prevalence rates of cyberbullying victimization. Since only one participant was born in 2002, the year 2002 was not included in the calculation of moving averages. In a next step, to compare the prevalence rates of cyberbullying victimization over the course of time, year of birth was dummy coded with years of birth 1980 to 1982 = 0, years of birth 1983–1985 = 1, years of birth 1986–1988 = 2, years of birth 1989–1991 = 3, years of birth 1992–1994 = 4, years of birth 1995–1997 = 5, years of birth 1998–2002 = 6. Because there were comparatively few cases born in 2001 and 2002, a larger range from 1998 to 2002 was chosen as the final age group. In order to examine the course of the moving averages in more detail, a trend analysis was carried out. For this purpose, a curve fitting procedure was utilized using linear, quadratic, and exponential regression models. In the next step, a logistic regression model was used to compare reports of cyberbullying victimization in patients born in all other years (1983 to 2002) to reports in patients born in the years 1980 to 1982. In addition, the model was controlled for effects of gender.

In order to determine the relative contribution of child maltreatment, traditional relational peer victimization, and cyberbullying victimization for the prediction of psychological symptom distress, a hierarchical multiple regression analysis was conducted. For this purpose, the continuous sum scores of the CTQ subscales emotional abuse, emotional neglect, physical abuse, and sexual abuse as well as the continuous modified sum score of the FBS were used. Additionally, the standardized (i.e., mean centred and divided by the standard deviation) cyberbullying victimization item in the adolescence scale of the FBS was used. In the hierarchical regression analysis, age and gender were included as predictors in a first step. In a second step, emotional abuse, emotional neglect, physical abuse, and sexual abuse were added. Traditional relational peer victimization was added in a third step. Finally, cyberbullying victimization in adolescence was entered in a fourth step. Preliminary analyses showed no violation of the assumption of multicollinearity (tolerances > 0.36; variance inflation factors < 1.28), linearity, normality, and homoscedasticity.

For exploratory purposes, additional ANCOVAs with child maltreatment and traditional relational peer victimization serving as covariates were used to compare victims of cyberbullying victimizations and those who did not report such victimization on the BSI subscales and the General Severity Index. All tests were adjusted using Bonferroni correction for multiple testing resulting in *p* = .05/10 = 0.005. Effect sizes were calculated using partial η^2^ [46]. According to Cohen [46], partial η^2^ values of 0.01 represent a small effect, 0.06 a medium effect, and 0.14 a large effect, respectively.

## Results

### Descriptive and preliminary analyses

Distribution of subject characteristics and mean values on the assessments are presented in Table 1. Preliminary bivariate analyses indicated positive intercorrelations between traditional relational peer victimization, cyberbullying victimization, and different forms of child maltreatment (Table 2). Additional bivariate analyses yielded positive correlations between different forms of peer victimization as well as different forms of child maltreatment and the different symptom dimensions as measured by the BSI subscales (Table 3).

**Table 2:** Intercorrelations between maltreatment forms, traditional relational peer victimization, and cyberbullying victimization in adolescence (N = 827)

	Emotional abuse	Physical abuse	Sexual abuse	Emotional neglect	Traditional relational peer victimization
Emotional abuse	1.00				
Physical abuse	0.64^***^	1.00			
Sexual abuse	0.33^***^	0.33^***^	1.00		
Emotional neglect	0.67^***^	0.48^***^	0.19^***^	1.00	
Traditional relational peer victimization	0.45^***^	0.29^***^	0.16^***^	0.32^***^	1.00
Cyberbullying victimization in adolescence	0.09^**^^*^	0.07^***^	0.13^***^	0.05	0.25^***^

Note. Correlations are represented by Pearson’s r for continuous predictor variables and point-biserial correlation for dichotomous predictor variables. ^*^p < .05; ^**^p < .01; ^***^p < .001.

**Table 3:** Intercorrelations between maltreatment forms, traditional relational peer victimization, cyberbullying victimization in adolescence and psychological symptom distress (N = 827)

	GSI	SOM	O-C	I-S	DEP	ANX	HOS	PHOB	PAR	PSY
Emotional abuse^1^	0.39^***^	0.22^***^	0.24^***^	0.33^***^	0.26^***^	0.28^***^	0.38^***^	0.22^***^	0.40^***^	0.30^***^
Physical abuse^1^	0.30^***^	0.15^***^	0.17^***^	0.21^***^	0.20^***^	0.22^***^	0.31^***^	0.20^***^	0.32^***^	0.22^***^
Sexual abuse^1^	0.13^***^	0.07^***^	0.07^***^	0.08^***^	0.09^***^	0.11^***^	0.11^***^	0.08^***^	0.14^***^	0.12^***^
Emotional neglect^1^	0.30^***^	0.14^***^	0.22^***^	0.25^***^	0.25^***^	0.16^***^	0.25^***^	0.16^***^	0.35^***^	0.26^***^
Traditional relational peer victimization^2^	0.40^***^	0.16^***^	0.32^***^	0.40^***^	0.36^***^	0.25^***^	0.28^***^	0.19^***^	0.37^***^	0.28^***^
Cyberbullying victimization in adolescence	0.20^***^	0.18^***^	0.14^***^	0.17^***^	0.10^**^	0.17^***^	0.16^***^	0.16^***^	0.19^***^	0.13^***^

Note: Correlations are represented by Pearson’s r for continuous predictor variables and point-biserial correlation for dichotomous predictor variables. SOM, somatization: O-C, obsessive-compulsivity: I-S, interpersonal sensitivity; DEP, depression: ANX, anxiety: HOS, hostility: PHOB, phobic anxiety: PAR, paranoid ideation: PSY, psychoticism; ^1^ assessment of childhood maltreatment including severity ratings; ^2^ Sum score of the FBS under exclusion of the physical and cyber item;^*^p < .05; ^**^p < .01; ^***^p < .001.

### Trend analyses of cyberbullying victimization over the past 20 years

**Fig. 1:**
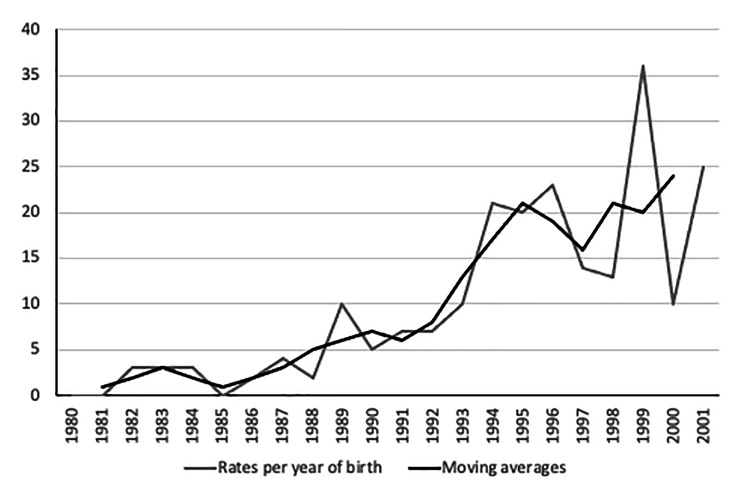
Rate of participants reporting cyberbullying victimization in adolescence per year of birth and moving averages taking the averages of three consecutive years of in % (*N* = 827). Since only one participant was born in 2002, this year was not included in the calculation of rates and moving averages

Overall, 8.3% of the patients born in the years 1980 to 2002 reported the experience of cyberbullying victimization in their adolescence. At the descriptive level, there is an increase in cyberbullying victimization over time (Fig. 1). The rate of reported cyberbullying victimization was low in patients born in the years 1980 to 1987 with moving averages ranging from 1 to 3%. For patients born after the year 1987 the moving averages increased consistently over time and was highest for patients born in the year 2000 (24%). In a next step, we utilized linear, quadratic, and exponential regression models to concurrently determine year of birth-related variations in moving averages. While all regression models fit the data and their determinant coefficients (*R*^2^) were of statistical significance (all *p*’s < 0.001), the exponential regression model fit best, i.e., *R*^*2*^ was maximized (linear: *R*^*2*^ = 0.79; quadratic: *R*^*2*^ = 0.79; exponential: *R*^*2*^ = 0.86).

A logistic regression was performed to ascertain the effects of year of birth (1980 to 2002) on the likelihood to report cyberbullying victimization when controlling for the effects of gender. The logistic regression model was statistically significant, χ² (8, *N* = 827) = 50.94, *p <* .001. The model explained 13.7% (Nagelkerke *R*2) of the variance in reporting cyberbullying victimization and correctly classified 91.7% of cases. Patients born in the years 1998–2002 were up to nineteen times as likely to report cyberbullying victimization as patients born in the years 1980–1982 (Table 4). While being born in the years 1983–1991 was not associated with a significantly increased likelihood of reporting cyberbullying victimization, being born in the years 1992–2002 was associated with a significant increased likelihood. In addition, being female was associated with an increased likelihood of reporting cyberbullying victimization.

**Table 4:** Logistic regression results for year of birth predicting the rate of reports of cyberbullying victimization when controlling for gender (N = 827)

	*ORs (95% Cis)*	*p*
Gender		
Male	1	
Female	1.66 (0.96 to 2.90)	0.072
Year of Birth		
1980–1982 (*n* = 79; 55.7% female)	1	
1983–1985 (*n* = 107; 67.3% female)	1.41 (0.13 to 15.82)	0.782
1986–1988 (*n* = 157; 55.4% female)	2.04 (0.22 to 18.61)	0.526
1989–1991 (*n* = 180; 60.6% female)	5.95 (0.76 to 46.35)	0.089
1992–1994 (*n* = 149; 63.8% female)	11.71 (1.54 to 89.09)	0.017
1995–1997 (*n* = 113; 60.2% female)	17.59 (2.31 to 133.94)	0.006
1998–2002 (*n* = 42; 57.1% female)	19.30 (2.31 to 161.0)	0.006

### Influence of adolescent cyberbullying victimization on psychological symptom distress

A gender- and age-adjusted hierarchical regression analysis was carried out for the Global Severity Index of the BSI to examine the unique contributions of different kinds of child maltreatment, traditional relational peer victimization, and cyberbullying victimization in the prediction of psychological symptom distress. Cyberbullying victimization made a significant incremental contribution of variance (1.1%) to the prediction of the score beyond the variance explained by child maltreatment and traditional relational peer victimization (Table 5). In the final model (*F*(8, 807) = 32.92, adjusted *R*2 = 0.24, *p* < .001), however, traditional relational peer victimization was the strongest predictor followed by emotional abuse and cyberbullying victimization.

**Table 5:** Hierarchical multiple regression for the prediction of psychological symptom distress (N = 827)

Variable	*Β*	SE	*β*	*p*	*R* ^2^	Δ*R*²	Δ*F*
Step 1					0.02	0.02	10.01^***^
Gender	0.12	0.04	0.09	0.003			
Age	− 0.01^*^	0.01	− 0.07^*^	0.034			
Step 2^1^					0.18	0.15	36.90^***^
Emotional abuse	0.02	0.01	0.14	0.006			
Physical abuse	0.02	0.01	0.09	0.027			
Sexual abuse	− 0.01	0.01	− 0.03	0.387			
Emotional neglect	0.01	0.01	0.09	0.025			
Step 3					0.24	0.06	64.10^***^
Traditional relational peer victimization^2^	0.02	0.01	0.25	< 0.001			
Step 4					0.25	0.01	11.53^***^
Cyberbullying victimization in adolescence	0.25	0.07	0.11	< 0.001			

Note: ^*^p < .05; ^**^p < .01; ^***^p < .001; β coefficients of the final models are presented; ^1^assessment of childhood maltreatment including severity ratings; ^2^Sum score of the FBS under exclusion of the physical and cyber item.

**Fig. 2:**
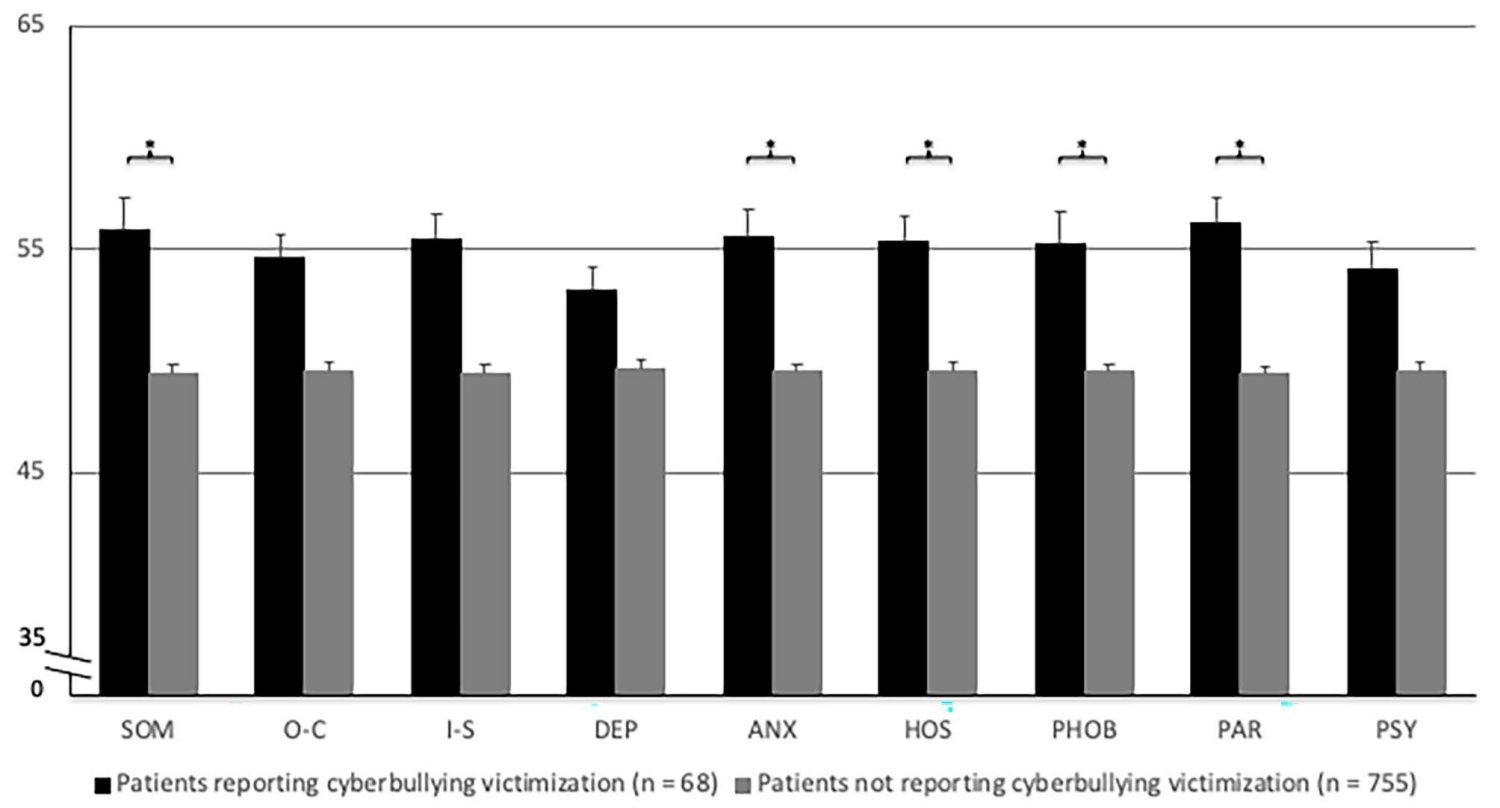
Normalized standard means (*t*-scores, standard error) for the comparison of participants reporting cyberbullying victimization and participants not reporting cyberbullying victimization on the BSI subscales. SOM, somatization; O-C, obsessive-compulsivity; I-S, interpersonal sensitivity; DEP, depression; ANX, anxiety; HOS, hostility; PHOB, phobic anxiety; PAR, paranoid ideation; PSY, psychoticism. *p < .05/10 when controlling for child maltreatment and traditional relational peer victimization

Additionally, exploratory ANCOVAs comparing cyberbullying victims and those not indicating cyberbullying victimization in their adolescence with child maltreatment and traditional relational peer victimization serving as covariates indicated higher scores for patients reporting cyberbullying on the BSI subscales somatization, anxiety, hostility, phobic anxiety, and paranoid ideation (see Fig. 2). The respective ANCOVAs showed significance (all *F*s > 8.89, all *p*s < 0.005) with small effect sizes ranging from *partial η*^*2*^ = 0.01 (anxiety) to *partial η*^*2*^ = 0.02 (somatization). For overall symptom distress as measured by the General Severity Index, a significant difference between groups was found (*F*(1, 812) = 13.46, *p* < .001, *partial η*^*2*^ = 0.016).

## Discussion

The present study sought to examine whether the increase of cyberbullying victimization over the last two decades is also reflected by enhanced reports of cyberbullying victimization in a German clinical outpatient population. In accordance with the emerging increase of cyberbullying victimization in student populations [10–12], elevated prevalence rates over a period of 20 years were also found in the present clinical population. The present data suggest that the increase can best be described as exponential. More specifically, younger patients born in the years 1995–2002 who are currently undergoing psychotherapeutic treatment were up to nineteen times as likely to retrospectively report cyberbullying victimization as older patients born in the years 1980–1982. Hence, the present findings emphasize the growing importance of this phenomenon not only in the general population but also in clinical care settings.

Comparing the present frequencies of cyberbullying victimization to reports of Kliem et al. [12] who reported 6-month prevalence rates of about 6% among German ninth graders in 2017, the present study revealed higher rates in outpatients of around 20% for patients born after the year 1995. The higher incidence rates of cyberbullying victimization in the present clinical sample align with existing literature with respect to findings that treatment-seeking patients are highly susceptible to experiencing various types of victimization throughout their lives [47] and report higher frequencies of traditional peer victimization [48]. However, prevalence rates of cyberbullying victimization found in the present study, at least for patients born after 1987, were higher when compared to an outpatient sample of children and adolescents examined by Kranhold et al. [29]. In their study, approximately 3% of patients reported cyberbullying victim or bully/victim status. These differences may be explained by different samples and methodological approaches (e.g., age range of the respondents, and instruments used to measure bullying). Hence, the present study indicates that the experiences of cyberbullying victimization in adolescence can also be recalled in adult clinical samples as compared to studies using student samples or younger outpatient samples whose victimization experiences are more recent. However, future studies are needed to investigate its distribution in different samples.

In addition to the increasing emergence of cyberbullying victimization in outpatients, the investigation of the unique contributions of cyberbullying victimization to psychopathology and its association to symptom severity underscore the importance of incorporating greater awareness of these experiences in research and clinical contexts. While Sansen et al. [48] revealed that traditional peer victimization represents an independent predictor for psychopathology when compared to different forms of child maltreatment, the present findings extend these findings by indicating that cyberbullying victimization in adolescence predicts psychological symptoms beyond the effects of both experiences of child maltreatment and traditional peer victimization. The significant incremental effect of cyberbullying victimization on psychopathology is consistent with previous studies with non-clinical samples illustrating that cyberbullying victimization uniquely contributes to psychological symptom distress (e.g., [19, 34, 35, 49]. Hence, our findings suggest that more variability in psychological symptom distress is explained when child maltreatment, traditional relational peer victimization, as well as cyberbullying victimization are assessed. It should be noted that the current effect size of the incremental effect of cyberbullying victimization is very small (1%). However, it is also of note that the current effect size is in line with effect sizes reported in previous studies. Giumetti and Kowalski [49], for example, reported small but significant amounts of variance explained in their outcomes of interest (absenteeism, anxiety, depression, grades, physical health, and self-esteem), ranging from 1 to 4%. Moreover, even when controlling for experiences of child maltreatment and traditional relational peer victimization, exploratory analyses indicated that outpatients who experienced cyberbullying victimization showed higher scores on various psychopathological symptom dimensions, such as somatization, anxiety, hostility, phobic anxiety, and paranoid ideation. However, contrasting with previous studies [15, 16], depression no longer differed between those who reported cyberbullying victimization and those who reported no victimization when controlling for other kinds of child maltreatment. Considering these findings, the present study indicated that cyberbullying victimization in adolescence in and of itself heightens the risk for the development of a broad range of psychopathology and therefore constitutes a relevant problem in its own right [31, 32].

### Strengths and limitations

The current study adds to the growing body of research highlighting the psychopathological outcomes of cyberbullying victimization. The generalizability of the present findings is strengthened through our use of a large treatment-seeking sample to determine prevalence rates and the relative importance of cyberbullying victimization for predicting psychopathology. However, the present study has several limitations. Notably, assessing cyberbullying victimization using one item in the FBS may have led to reduced reliability and validity [50]. Studies comparing different assessments of prevalence rates of cyberbullying victimization reported rather conservative rates when using single item assessments [51]. Hence, it is likely that rates of cyberbullying victimization among patients with mental disorders are even larger than reported in the present study – again highlighting its relevance for clinical research and treatment. Moreover, since the questionnaire does not explicitly emphasize the aspect of repetition (as emphasized in the definition of cyberbullying), it remains unclear whether recalled experiences indeed represent cyberbullying victimization [52]. Nevertheless, the implemented questionnaire was useful to gain an initial overview on cyber victimization experiences in a large clinical sample and over a long period of time as the phenomenon of cyberbullying victimization has rarely been studied in clinical treatment-seeking samples (e.g., [29, 53]). Additionally, due to the relative novelty of this phenomenon, research has not yet agreed upon a fixed definition of the cyberbullying construct. The lack of agreement in the field over what constitutes cyberbullying makes it difficult to compare results from different studies and countries.

Further, due to the predefined dataset, only cross-sectional analyses could be performed. Thus, causal conclusions cannot be made. Moreover, associations and interactions between different forms of child maltreatment, traditional peer victimization, and cyberbullying victimization and its time courses were not fully examined in the present study. There is evidence that cyberbullied adolescents are likely to be exposed to multiple forms of victimization in other environments as well or even put individuals at risk for experiencing cyberbullying victimization in adolescence (e.g., [4, 30, 54–59]). However, on the basis of the available data, it was not possible to analyse these interacting courses of adverse life experiences. The retrospective assessment of victimization also implies a limitation to the study. Retrospective accounts may be subject to recall biases [60]. However, there is evidence that recall biases in the assessment of childhood maltreatment were not large enough to invalidate retrospective reports [61]. In addition, retrospective reports of emotional forms of maltreatment lack valid alternatives, since they are not reliably documented in child protection service, clinical, or medical records. In addition, in the present study child maltreatment was assessed using severity ratings whereas traditional relational peer victimization and cyberbullying victimization were measured using an event checklist indicating whether participants have experienced aversive situations or not. The difference in the questions in each instruments could have had an impact on the results. Future studies should use instruments that uniformly capture maltreatment experiences.

### Clinical implications

Repeated experiences of cyberbullying victimization reflect an additional type of maltreatment that surpasses the influence of child maltreatment and traditional relational peer victimization on the development of psychopathology. Therefore, the potential effects of exposure to cyberbullying victimization, by itself and in combination with other forms of maltreatment, need to be carefully considered in future research studies. For instance, our findings suggest that different combinations of on- and offline maltreatment types may lead to varying profiles in psychopathology. As a consequence, these profiles may have differential effects on the formation of relationships. Therefore, future studies could investigate the extent to which cyberbullying, as opposed to other forms of maltreatment, affects social life off- and online.

Furthermore, mental health professionals (e.g., psychotherapists, physicians, and diagnosticians) should actively inquire, perceive, and communicate the consequences of cyberbullying victimization to patients. This is of particular importance as experiences of cyberbullying can be involved in the development and maintenance of a range of mental and physical illnesses. Moreover, treatment of psychiatric disorders resulting from cyberbullying victimization should include therapeutic interventions that combine symptom- and disorder-specific treatment approaches with interventions that focus on reprocessing traumatic life experiences, including cyberbullying victimization. Finally, our findings on the consequences of cyberbullying victimization emphasize that this form of maltreatment should addressed more prominently as a societal risk factor in order to prevent mental disorders and identify children at risk.

## Conclusion

In conclusion, this retrospective survey represents a valuable first insight into some of the long-term consequences of cyberbullying victimization. Prevalence rates and the unique contribution to general symptom distress underscored the increasing problem of cyberbullying victimization, indicating that such victimization is a particularly serious concern that requires attention in both prevention and intervention in the mental health care system. However, due to methodological constraints, further clinical samples are needed to verify the results of this study.

## Data Availability

The datasets used and/or analysed during the current study are available from the corresponding author on reasonable request.

## References

[1] Hajok D (2019). Der veränderte Medienumgang Jugendlicher. Tendenzen aus 20 Jahren JIM-Studie. JMS Jugend Medien Schutz-Report.

[2] Reid D, Weigle P (2014). Social media use among adolescents: benefits and risks. Adolesc Psychiatry (Hilversum).

[3] Buchanan R, Scevak J, Smith S, Southgate E. Disclosure in the postdigital age: university students’ attitudes to social media. In: 6th European Conference on Social Media (ECSM 2019). Proceedings of the 6th European Conference on Social Media (ECSM 2019). Academic Conferences and Publishing International; 2019. p. 58–64.

[4] Craig W, Boniel-Nissim M, King N, Walsh SD, Boer M, Donnelly PD (2020). Social Media Use and Cyber-Bullying: a cross-national analysis of Young People in 42 countries. J Adolesc Heal.

[5] Tokunaga RS (2010). Following you home from school: a critical review and synthesis of research on cyberbullying victimization. Comput Hum Behav.

[6] Johansson S, Englund G (2021). Cyberbullying and its relationship with physical, verbal, and relational bullying: a structural equation modelling approach. Educ Psychol.

[7] Smith PK (2015). The nature of cyberbullying and what we can do about it. J Res Spec Educ Needs.

[8] Slonje R, Smith PK, Frisén A (2013). The nature of cyberbullying, and strategies for prevention. Comput Hum Behav.

[9] Suler J (2004). The online disinhibition effect. Cyberpsychology and Behavior.

[10] Jones LM, Mitchell KJ, Finkelhor D (2013). Online harassment in context: Trends from three youth internet safety surveys (2000, 2005, 2010). Psychol Violence.

[11] Kessel Schneider S, O’Donnell L, Smith E (2015). Trends in Cyberbullying and School bullying victimization in a Regional Census of High School Students, 2006–2012. J Sch Health.

[12] Kliem S, Krieg Y, Baier D (2020). Allgemeine und spezifische Entwicklung von Cybermobbing unter Jugendlichen: Ergebnisse aus repräsentativen Befragungen unter niedersächsischen Schülerinnen und Schülern. Kindh Entwickl.

[13] Pontes NMH, Ayres CG, Lewandowski C, Pontes MCF (2018). Trends in bullying victimization by gender among U.S. high school students. Res Nurs Health.

[14] Kowalski RM, Limber SP (2013). Psychological, physical, and academic correlates of cyberbullying and traditional bullying. J Adolesc Heal.

[15] Perren S, Dooley J, Shaw T, Cross D (2010). Bullying in school and cyberspace: Associations with depressive symptoms in swiss and australian adolescents. Child Adolesc Psychiatry Ment Health.

[16] Wang J, Nansel TR, Iannotti RJ (2011). Cyber and traditional bullying: Differential association with depression. J Adolesc Heal.

[17] Brighi A, Melotti G, Guarini A, Genta ML, Ortega R, Mora-Merchán J et al. Self-esteem and loneliness in relation to Cyberbullying in three european countries. Cyberbullying in the global playground: research from International Perspectives. Wiley-Blackwell; 2012. 32–56.

[18] Juvonen J, Gross EF (2008). Extending the School Grounds?-Bullying Experiences in Cyberspace. J Sch Health.

[19] Wigderson S, Lynch M (2013). Cyber-and traditional peer victimization: unique relationships with adolescent well-being. Psychol Violence.

[20] Sourander A, Klomek AB, Ikonen M, Lindroos J, Luntamo T, Koskelainen M (2010). Psychosocial risk factors associated with cyberbullying among adolescents: a population-based study. Arch Gen Psychiatry.

[21] Hinduja S, Patchin JW (2010). Bullying, cyberbullying, and suicide. Arch Suicide Res.

[22] Messias E, Kindrick K, Castro J (2014). School bullying, cyberbullying, or both: correlates of teen suicidality in the 2011 CDC youth risk behavior survey. Compr Psychiatry.

[23] Mitchell KJ, Ybarra M, Finkelhor D (2007). The relative importance of online victimization in understanding depression, delinquency, and substance use. Child Maltreat.

[24] Ybarra ML, Diener-West M, Leaf PJ (2007). Examining the overlap in internet harassment and school bullying: implications for school intervention. J Adolesc Heal.

[25] Fisher BW, Gardella JH, Teurbe-Tolon AR (2016). Peer cybervictimization among adolescents and the Associated Internalizing and externalizing problems: a Meta-analysis. J Youth Adolesc.

[26] Nixon C (2014). Current perspectives: the impact of cyberbullying on adolescent health. Adolesc Health Med Ther.

[27] Nicolai S, Geffner R, Stolberg R, Yaruss JS (2018). Retrospective experiences of cyberbullying and emotional outcomes on young adults who Stutter. J Child Adolesc Trauma.

[28] Scheithauer H, Petras IK, Petermann F, Cybermobbing (2020). / Cyberbullying Kindheit und Entwicklung.

[29] Kranhold AL, Voigt B, Wolke D, Krause K, Friedrich S, Margraf J (2021). Bullying experiences in outpatients of a child and adolescent psychotherapy centre-A particularly vulnerable group?. Z Kinder Jugendpsychiatr Psychother.

[30] Modecki KL, Minchin J, Harbaugh AG, Guerra NG, Runions KC (2014). Bullying prevalence across contexts: a meta-analysis measuring cyber and traditional bullying. J Adolesc Health.

[31] Olweus D (2012). Cyberbullying: an overrated phenomenon?. Eur J Dev Psychol.

[32] Smith PK, Cyberbullying (2012). Challenges and opportunities for a research program-A response to Olweus (2012). Eur J Dev Psychol.

[33] Hase CN, Goldberg SB, Smith D, Stuck A, Campain J (2015). Impacts of traditional bullying and cyberbullying on the mental health of middle school and high school students. Psychol Sch.

[34] Fredstrom BK, Adams RE, Gilman R (2011). Electronic and School-Based victimization: unique contexts for Adjustment Difficulties during Adolescence. J Youth Adolesc.

[35] Gini G, Card NA, Pozzoli T (2018). A meta-analysis of the differential relations of traditional and cyber-victimization with internalizing problems. Aggress Behav.

[36] Van Geel M, Vedder P, Tanilon J (2014). Relationship between peer victimization, cyberbullying, and suicide in children and adolescents ameta-analysis. JAMA Pediatr.

[37] Benedini KM, Fagan AA, Gibson CL (2016). The cycle of victimization: the relationship between childhood maltreatment and adolescent peer victimization. Child Abus Negl.

[38] Espelage DL, Low S, De La Rue L (2012). Relations between peer victimization subtypes, family violence, and psychological outcomes during early adolescence. Psychol Violence.

[39] Tremblay-Perreault A, Hébert M (2020). Uncovering the Associations between child sexual abuse, peer victimization and behavior problems using child, parent and teacher reports. J Sch Violence.

[40] Yoon D, Yoon S, Park J, Yoon M (2018). A pernicious cycle: finding the pathways from child maltreatment to adolescent peer victimization. Child Abus Negl.

[41] Sansen LM, Iffland B, Catani C, Neuner F (2013). Entwicklung und evaluation des Fragebogens zu belastenden Sozialerfahrungen in der Peergroup (FBS)[Development and evaluation of a questionnaire on stressful social experiences in peer groups (FBS)]. Z Klin Psychol Psychother.

[42] Willard NE. Cyberbullying and cyberthreats: responding to the challenge of online social aggression, threats, and distress. Research Press; 2007.

[43] Wingenfeld K, Spitzer C, Mensebach C, Grabe HJ, Hill A, Gast U (2010). Die deutsche Version des Childhood Trauma Questionnaire (CTQ): erste Befunde zu den psychometrischen kennwerten [The german version of the Childhood Trauma Questionnaire (CTQ): preliminary psychometric properties]. Psychother Psych Med.

[44] Klinitzke G, Romppel M, Häuser W, Brähler E, Glaesmer H (2012). The german version of the Childhood Trauma Questionnaire (CTQ): psychometric characteristics in a representative sample of the general population. Psychother Psychosom Med Psychol.

[45] Franke G (2000). BSI - brief Symptom-Inventory von L.R. Derogatis. Deutsche Version. Manual.

[46] Cohen J. Statistical power analysis for the behavioral sciences. In: Statistical Power Analysis for the Behavioral Sciences. 1988.

[47] Brady KL, Caraway SJ (2002). Home away from home: factors associated with current functioning in children living in a residential treatment setting. Child Abus Negl.

[48] Sansen LM, Iffland B, Neuner F (2014). Peer victimization predicts psychological symptoms beyond the effects of child maltreatment. Psychiatry Res.

[49] Giumetti GW, Kowalski RM, Li Q, Cross D, Smith PK (2016). Cyberbullying matters: examining the incremental impact of cyberbullying on outcomes over and above traditional bullying in North America. Cyberbullying in the global playground: research from International Perspectives.

[50] Yanagida T, Gradinger P, Strohmeier D, Solomontos-Kountouri O, Trip S, Bora C (2016). Cross-national prevalence of traditional bullying, traditional victimization, cyberbullying and cyber-victimization: comparing single-item and multiple-item approaches of measurement. Int J Dev Sci.

[51] Zych I, Ortega-Ruiz R, Marín-López I, Cyberbullying (2016). A systematic review of research, its prevalence and assessment issues in spanish studies. Psicol Educ.

[52] Hunter SC, Boyle JME, Warden D (2007). Perceptions and correlates of peer-victimization and bullying. Br J Educ Psychol.

[53] Stecher N, Bock A, Fleischmann S, Fuchs M, Bliem HR, Juen B (2019). Prevalence and characteristics of peer victimisation in adolescent psychiatric inpatients. Z Kinder Jugendpsychiatr Psychother.

[54] Turner HA, Finkelhor D, Ormrod R (2010). Poly-victimization in a National Sample of Children and Youth. Am J Prev Med.

[55] Lereya ST, Samara M, Wolke D (2013). Parenting behavior and the risk of becoming a victim and a bully/victim: a meta-analysis study. Child Abus Negl.

[56] Kennedy RS, Font SA, Haag AC, Noll JG (2021). Childhood sexual abuse and exposure to peer bullying victimization. J Interpers Violence.

[57] Hébert M, Cénat JM, Blais M, Lavoie F, Guerrier M (2016). Child sexual abuse, bullying, cyberbullying, and mental health problems among high schools students: a moderated mediated model. Depress Anxiety.

[58] Widom CS. Longterm consequences of child maltreatment. Child maltreatment: Contemporary Issues in Research and Policy. Springer Nature; 2014. 225–47.

[59] Worsley JD, McIntyre JC, Bentall RP, Corcoran R (2018). Childhood maltreatment and problematic social media use: the role of attachment and depression. Psychiatry Res.

[60] Häuser W, Schmutzer G, Brähler E, Glaesmer H (2011). Maltreatment in childhood and adolescence—results from a survey of a representative sample of the german population. Dtsch Arztebl Int.

[61] Hardt J, Rutter M (2004). Validity of adult retrospective reports of adverse childhood experiences: review of the evidence. J Child Psychol Psychiatry.

